# Supplementary material for: Splice-Junction-Based Mapping of Alternative Isoforms in the Human Proteome
Source: Cell Rep. Author manuscript; Available in PMC 2020 Jan 15. (PMC6961840; doi:10.1016/j.celrep.2019.11.026)
Supplement: 2 [file NIHMS1546469-supplement-2.zip › DF1/PXD010154/AdrenalGland/AdrenalGland_15_SRSF1_ALGLVGRPVLESALFVTAMSGGGVIR.pdf]

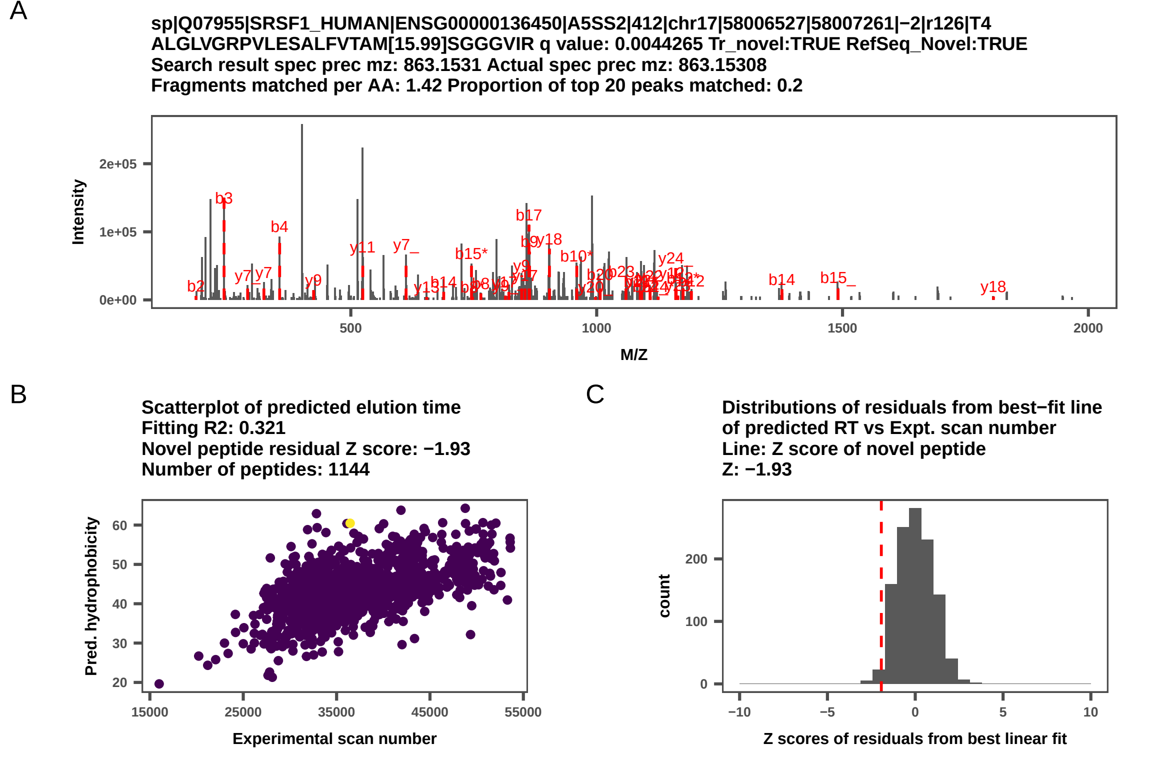
A
sp|Q07955|SRSF1_HUMAN|ENSG00000136450|A5SS2|412|chr17|58006527|58007261|−2|r126|T4
ALGLVGRPVLESALFVTAM[15.99]SGGGVIR q value: 0.0044265 Tr_novel:TRUE RefSeq_Novel:TRUE
Search result spec prec mz: 863.1531 Actual spec prec mz: 863.15308
Fragments matched per AA: 1.42 Proportion of top 20 peaks matched: 0.2
Intensity
2e+05
1e+05
0e+00
M/Z
500
1000
1500
2000
b2
b3
b4
y7_
y7
y9
y11
y7_
b15*
b14
b17
b9
y18
b10*
y24
b14
b15_
y18
B
Scatterplot of predicted elution time
Fitting R2: 0.321
Novel peptide residual Z score: −1.93
Number of peptides: 1144
Pred. hydrophobicity
60
50
40
30
20
Experimental scan number
15000
25000
35000
45000
55000
C
Distributions of residuals from best−fit line
of predicted RT vs Expt. scan number
Line: Z score of novel peptide
Z: −1.93
count
200
100
0
Z scores of residuals from best linear fit
−10
−5
0
5
10
